# Exfoliated kidney cells from urine for non-invasive kidney transplant monitoring: A potential opportunity?

**DOI:** 10.1007/s10157-026-02827-8

**Published:** 2026-02-09

**Authors:** Henry H. L. Wu, Naveen Kumar Parthiban, Ewa M. Goldys, Carol A. Pollock, Sonia Saad

**Affiliations:** 1 Renal Research, Kolling Institute of Medical Research, Royal North Shore Hospital and The University of Sydney, Kolling Building, St. Leonards, Sydney, NSW 2065 Australia; 2 Department of Renal Medicine, Royal North Shore Hospital, Northern Sydney Local Health District, Sydney, Australia; 3 ARC Centre of Excellence for Nanoscale Biophotonics, School of Biomedical Engineering, The University of New South Wales, Sydney, Australia

**Keywords:** Urinary biomarkers, Urinary exfoliated kidney cells, Kidney transplantation, Transplant monitoring, Non-invasive

## Abstract

Kidney transplantation is usually the optimal treatment option for patients living with kidney failure given its associations with improved survival, quality of life outcomes and a reduction in the personal, economic, and societal burden of long-term dialysis. While advantages of kidney transplantation are recognized, post-transplant complications, such as graft rejection, ischemia–reperfusion injury, surgical-related complications, and long-term consequences of immunosuppressive therapies, are commonly observed. There has been increased research on developing non-invasive biomarkers for the monitoring of transplanted kidneys over recent decades. The potential of urinary biomarkers to identify graft rejection, post-transplant acute tubular necrosis, detect progression of epithelial-to-mesenchymal transition toward tubulointerstitial fibrosis, and to differentiate between causes of graft dysfunction is an attractive alternative to invasive transplant biopsy. Innovative urinary biomarkers, such as those derived from omics technologies allow for a more holistic assessment of graft status through multi-parametric molecular analysis, although there remain questions on the consistency, reliability, and practicality of utilizing omics-based urinary biomarkers. The international nephrology community has continued to make concerted efforts to improve the procedures and cost-effectiveness of kidney transplant monitoring. In this article, we review the evidence and limitations of currently available urinary biomarkers and propose the application of urine-derived exfoliated kidney cells such as urinary exfoliated proximal tubule cells to prognosticate kidney transplant outcomes and monitor for post-transplant complications. Artificial intelligence and the incorporation of machine learning analysis of proximal tubular cell characteristics may optimize the process of differentiating graft rejection from other forms of kidney dysfunction non-invasively following kidney transplantation.

## Introduction

Kidney transplantation is the kidney replacement therapy of choice for eligible patients with kidney failure. When compared to dialysis over the long term, kidney transplantation has been associated with superior outcomes, notably in terms of survival and health-related quality of life [1]. Over the past decade in Australia, the Australian and New Zealand Dialysis and Transplant Registry (ANZDATA) highlighted a growth of 21% of kidney transplants performed between 2014 and 2019 [2]. Although the number of kidney transplant recipients has reduced since due to the COVID-19 pandemic, this has now returned back to pre-pandemic levels [3].

While the benefits of kidney transplantation are recognized, graft rejection occurring following transplant, ischemia–reperfusion injuries, hypoxia-induced acute tubular necrosis (ATN), immunosuppression-associated toxicities, and other post-surgical complications are frequently reported [4–6]. Close monitoring and regular follow-up of patients following kidney transplantation form an important part of post-transplant care. Guidelines established by consensus groups such as Kidney Disease Improving Global Outcomes (KDIGO) inform post-transplant monitoring and clinical management [7].

The gold standard investigation to determine the cause of unexplained serum creatinine rise post-transplant would usually be histological examination following an ultrasound-guided kidney biopsy [7]. However, the disadvantages of kidney biopsy are well-known. Hospitalization may be required and post-biopsy complications, such as bleeding and excess pain, are commonly described, and there may be a need for extended hospitalization if these complications are severe [8–10]. The stress of performing kidney biopsies routinely for the clinician has emerged with greater recognition, with the increasing workload and time pressures of the modern-day clinical environment [11]. There is currently a debate whether a non-invasively obtained biomarker could replace the need for routine kidney biopsy in these circumstances [12].

According to the United States National Institute of Health Biomarker Definition Working Group, an ideal biomarker in kidney transplantation should rapidly, accurately, inexpensively, and non-invasively identify subjects with incipient allograft injury, and discern the type of injury [13]. Clinically useful assays for such biomarkers have a high sensitivity and specificity, negative and positive predictive value, and an area under the receiver operator curve (AUC) well in excess of 0.5 and ideally nearing 1.0 [14]. While there is increasing discussion to what extent imaging and serum biomarkers can present as reliable options to accurately determine graft status, the development of urinary biomarkers as a prognostic tool in kidney transplantation presents another non-invasive option, and one that could cause less inconvenience for patients as it avoids the requirement of drawing blood and the need for special arrangements (i.e., tubes/kits) for collection [15]. In this article, we review the updated evidence surrounding urinary biomarkers in kidney transplantation and explore the potential of utilizing urinary exfoliated kidney cells in this context.

## Emerging diagnostic and prognostic urinary biomarkers to determine kidney transplant graft function and survival

Urine is a readily accessible biofluid and a direct ultrafiltrate of the kidneys which may be able to provide insightful information into graft status [16]. Traditional urinary biomarkers such as albuminuria or proteinuria are established in current guidelines for use as a screening tool to assess kidney transplant graft function [7]. However, its sensitivity and specificity are unlikely to be comparable to histological examination which remains the gold standard [17]. There are realizations that a single ‘perfect’ urinary marker in kidney transplantation is unlikely to exist, but efforts in search of a non-invasive option to monitor kidney transplants have continued (Table 1) [7–10, 12, 17–33].

**Table 1:** Comparative overview of diagnostic and prognostic tools in determining kidney transplant function and survival

Diagnostic/prognostic tool	Primary diagnostic targets	Representative readouts	Strengths	Limitations	References
Kidney biopsy	Etiology-resolving diagnosis (Banff categories etc.)	Histopathology	Highest diagnostic specificity; reference gold standard	Invasive; complications; sampling error; unsuitable for frequent monitoring	[8–10]
Serum creatinine and eGFR	Global transplant graft function	Trend over time	Widely available; low cost; standard of care	Etiology non-specific; results may lag behind actual histopathological progression; cannot discriminate ATN versus graft rejection versus drug toxicity to transplant graft	[7]
Proteinuria/albuminuria	Chronic transplant graft injury risks; association with glomerular and tubulointerstitial pathology in graft	ACR/PCR; longitudinal trend	Simple and scalable; can be readily applied as a prognostic marker for graft survival	Still not etiology-specific; affected by presence of infection and hemodynamic status;	[17]
Urinary extracellular vesicle cargo	Pathway-level signals; phenotype of allograft rejection and/or graft injury	EVsmRNA/protein signatures	Potentially information-rich; active transplant research area	Pre-analytics and isolation methods vary; needs harmonization and external validation	[18–20]
Urinary donor-derived cfDNA	Injury-associated nucleic acids; diagnosis and prognostication of allograft rejection and/or graft injury	Donor-derived cfDNA preservation/assay workflow	Non-invasive	Evidence base is still emerging; standardization of collection/preservation critical	[21]
Urinary KIM-1	Detects early proximal tubule and ischemia-perfusion damage in kidney transplantation	KIM-1 expression very low in healthy kidneys, expression dramatically increases in kidney injury	Non-invasive; highly sensitive biomarker; able to predict early graft injury as well as long-term graft survival	Still requires continuous validation as a consistently reliable marker to detect graft injury	[22–24]
Omics—urinary injury proteins	Tubular injury; early graft dysfunction phenotypes	NGAL, IL-18 (and panels)	Can determine biological process of graft injury; feasible assays	Marker specificity varies; may need combination models; confounding by infection/inflammation	[12, 25]
Omics—urinary chemokines	Immune activation; rejection-associated inflammation	CXCL9/CXCL10 (± creatinine ratio)	High sensitivity and specificity to identify graft rejection; non-invasive immune signal	Performance may vary dependent on analysis platform used; can be elevated in infection (e.g., BK virus)	[26, 27]
Omics—urinary mRNA/gene expression signatures	Acute cellular rejection and inflammation signatures	Multi-gene panels; urinary mRNA profiles	Biologically interpretable; validated multi-center evidence exists	Processing standardization required; operational complexity	[28, 29]
Imaging of urinary exfoliated kidney cells (i.e., machine learning analysis of imaged urinary exfoliated PTCs auto-fluorescence signals)	Tubular epithelial phenotype; demonstrated potential for accurate, non-invasive diagnosis of post-transplant ATN, graft rejection and IFTA	Optical signatures + machine learning classifier	Direct sampling of renal epithelial compartment; non-invasive	Current studies are proof-of-concept; requires multi-center validation	[30–33]

Over the past decade, innovation in the area of urinary biomarkers and subsequent development of precision assessment methods for graft dysfunction have been associated with the utilization of urinary exfoliated extracellular vesicles (EV) [19, 20]. EVs, being lipid bilayer–delimited particles with unique biosignatures and immunomodulatory function, act as intermediaries of cell signaling and possess excellent potential as a precise and non-invasive prognostic marker in kidney transplantation [19, 20, 34]. Specifically, urinary exfoliated EVs have demonstrated tremendous efficacy as a tool to assess donor kidney quality and graft function pre-transplantation, determine risk of kidney allograft rejection post-transplantation, and as a therapeutic tool to modulate post-transplant alloimmune responses and to treat graft ischemia–reperfusion injuries [19]. Ongoing work to validate the utility of urinary exfoliated EVs within the kidney transplantation context is anticipated.

Otherwise, urinary donor-derived cell-free deoxyribonucleic acid (cfDNA) provides an orthogonal view into graft injury and may also better capture tubular injury, immune activation and microenvironmental signals. Recent studies demonstrated that urinary donor-derived cfDNA can monitor transplant graft rejection (especially antibody-mediated rejection) and acute graft injury with high sensitivity, outperforming serum creatinine in early detection of kidney transplant complications [21, 35, 36]. Typically, urinary cfDNA > 1% is the threshold indicating active graft rejection [37]. However, studies investigating this biomarker are limited by uncertainties due to the source of extracted cfDNA (i.e., whether urinary cfDNA originated from a donor kidney or not, and whether urinary cfDNA is derived from a deceased or a living donor), heterogeneous analysis platforms, lack of standardization in urine processing (timing, centrifugation, storage, and normalization), and the relative paucity of large, prospectively designed transplant cohorts compared with serum-based biomarker assays [19–21, 34, 38].

Measuring urinary kidney injury molecule 1 (KIM-1) level is another approach that has been considered. KIM-1 is a highly sensitive biomarker which can detect early proximal tubule and ischemia-perfusion damage in kidney transplantation, offering a non-invasive tool to assess donor injury (especially brain-dead donors) and predict long-term graft survival [22, 23]. It is increasingly established that urinary excretion of KIM-1 is an independent predictor of long-term graft loss and a promising novel biomarker for early prediction of graft loss, with urinary KIM-1 levels outperforming serum creatinine in terms of diagnostic accuracy within this context [24].

Beyond these emerging urinary biomarkers as outlined in the previous section, the revolutionary innovation of high-throughput omics technologies allows for a comprehensive assessment of omics markers and through a multi-parametric molecular approach, addresses the holistic complexity of the pathophysiological mechanisms underlying transplant graft outcomes [39–41]. Numerous urinary omics markers could be utilized throughout a donor and patient’s transplantation journey to prognosticate outcomes—before procurement, preservation of the donor kidney, and after transplantation [26–29, 39, 42–68]. Omics markers have the potential to anticipate both short- (e.g., acute T cell/antibody-mediated rejection) and long-term outcomes (e.g., chronic allograft rejection), and also augment knowledge regarding graft adaptation and graft function post-transplantation [39]. Omics techniques hinge on an algorithmic methodology often involving machine learning applications to achieve their objectives [39, 40].

Numerous genomic-, proteomic-, transcriptomic-, and metabolomic-based signatures have been investigated for non-invasive diagnosis of kidney transplant graft rejection (Table 2) [26–29, 39, 42–68]. Findings from both genomics and transcriptomics analysis noted CXCL10 (IP-10) messenger ribonucleic acid (mRNA) demonstrating high sensitivity (100%) for graft rejection (although only with 78% specificity) [56, 67]. Proteomics analysis also identified urinary CXCL9 and CXCL10 with high sensitivity (86.4%) and specificity (91.3%) for graft rejection [26]. Urinary chemokines (e.g., CXCL9, CXCL10) have now been interrogated in large cohort and multi-center studies as well as systematic reviews, and have shown tremendous promise as diagnostic biomarkers for acute graft rejection [15, 18, 26, 42, 43, 69, 70]. Published studies relating to urinary metabolomics analysis noted kynurenine, proline, and a combined metabolomic biomarker comprising of mRNA signatures and kynurenine having a good level of sensitivity and specificity for events of transplant graft rejection [29, 44].

**Table 2:** Examples of urinary ‘omics’ markers utilized for monitoring of the transplanted kidney(s)

Type of omics	Omics marker	Sensitivity (%)/Specificity (%) for events of transplant rejection
Genomics [47, 67]	CD103 mRNA	59/75
	CXCL10 (IP-10) mRNA	100/78
	CXCR mRNA	63/83
Proteomics [26, 27, 42, 43, 45, 48–50, 52, 60–62, 64, 65]	ANXA11	NA
	β2-microglobulin	83.3/80
	β-Defensin-1/α1-antichymotrypsin	NA
	C4d	NA
	CXCL9	83/84
	CXCL9: Creatinine	86.4/91.3; 86/80; 86/64; 93/89; 81.2/34.5
	CXCL10	86.4/91.3
	CXCL10: Creatinine	80/76; 68/90; 77/60; 81.6/50.8
	Fractalkine	74.4/75
	Integrin α3	NA
	Integrin β3	NA
	NGAL	90/91
	TNF-α	NA
	sVCAM	NA
	Marker from a combined nine urine proteins (HLA class II protein HLA-DRB1, KRT14, HIST1H4B, FGG, ACTB, FGB, FGA, KRT7, DPP4)	NA
Transcriptomics [27, 28, 41, 51, 53–58, 62, 63, 66, 68]	CD103	59/75
	CXCL10 (IP-10)	NA; NA; 100/78
	CXCR-3	63/83
	Fox P3	90/73
	Granulysin	80/100; 96/67
	Granzyme A	80/100
	Granzyme B	79/77; 88/79; 60/100
	miR-210	52/74
	NKG2D	77/81
	Perforin	83/83; 88/79
	Serine proteinase inhibitor 9	76/79
	Tim-3	NA; 84/96
	Marker from a combination of mRNA for OX40, OX40L, PD-1 and Fox P3	95/92
	Marker from a 3-gene signature (18S ribosomal mRNA, CD3ε mRNA and CXCL10 mRNA)	79/78
	Marker from a 6-gene signature (CD3ε, CD105, CD14, CD46 and 18S rRNA)	NA
Metabolomics [29, 44]	Kynurenine	83/83
	Proline	83/83
	mRNA signature + 1.1164 × log(3-sialyllactose/xanthosine) kynurenine	82/87

Despite the attractions and advantages of omics technologies, there remains uncertainty as to the techniques that are consistent enough to obtain a complete profile of graft status. Over the years, scoring systems combining various non-omics urinary biomarkers (including proteinuria, cfDNA, methylated cfDNA, KIM-1, neutrophil gelatinase-associated lipocalin (NGAL), L-type fatty acid-binding protein and clusterin), and omics markers have been constructed for diagnosis and prediction of kidney transplantation outcomes, with numerous scoring systems achieving a high degree of diagnostic and prognostic accuracy [15, 69, 70]. Nevertheless, the use of these combined urinary biomarkers within the kidney transplantation context remains to be lacking in validation, rigorous design and methodology, accurate interpretation, and transparency [71]. Furthermore, translating laboratory-based biomarker assessments into interpretable diagnostic results indicating clinical action remains a time-consuming process, limiting its practicality in day-to-day practice [72]. In clinical practice, these techniques are likely deemed expensive and unaffordable in some settings [73]. More initiatives are needed to address the practicalities and challenges surrounding cost-effectiveness of integrating novel urinary biomarkers for kidney transplant monitoring.

## Utilizing urinary exfoliated kidney cells for kidney transplant monitoring

Cell exfoliation is a natural process where external cells are removed from the epithelial luminal surface to ensure the epithelium remains structurally intact and primed for further growth [74]. Exfoliation is part of homeostatic processes in mammalian organs, such as gut and placenta, as well as the kidney [74], considered to play a significant role in preserving the epithelial layer’s architectural integrity. Cells exfoliated into urine are most likely a single cell, or a group of cells from the epithelial layer which can be detached from tissue [74]. Small numbers of senescent epithelial cells are usually observed in exfoliated cells from urine of healthy individuals [75]. On the other hand, increasing amounts of exfoliated cells from urine (a mixture of both active and senescent cells) are expected in individuals with active kidney disease [75]. Given the passage of urine prior to excretion, some of these exfoliated cells would be sourced from the nephron, and can generate useful information regarding the kidney’s histopathological status [76]. Urinary exfoliated kidney cells have been considered over the past 30 years as a potentially useful non-invasive source in chronic kidney disease (CKD) for early diagnosis [77]. Urinary exfoliated PTCs, podocytes and kidney stem cells have been discussed within this context [30, 76, 78, 79]. Specifically, the number of exfoliated PTCs has been investigated as a measure of pathological status in the kidneys given proximal tubules make up majority of the kidney mass [74, 80, 81]. It has also been noted that the number of exfoliated PTCs correlate with kidney functional decline in CKD [81, 82].

Following an initial report of cell culture success from urine in newborn children in 1972 by Sutherland and Bain, cell culture techniques have been utilized in research to isolate urinary exfoliated kidney cells from urine [83]. Difficulties in the maintenance of urinary exfoliated kidney cells are acknowledged as mature exfoliated cells have a short lifespan in culture [84]. Immortalization techniques play an instrumental role in culture to extend cell survival. Biomedical technological advances have led to improved processes of cell isolation and characterization for urinary exfoliated kidney cells [75, 85, 86]. Numerous cell isolation and cell characterization techniques have been introduced over recent years, allowing urinary exfoliated kidney cells to be used as surrogate markers for biopsied tissue in predicting changes relating to gene expression, DNA methylation, DNA damage and protein expression [82, 84, 87–89]. Nevertheless, the utility of urinary exfoliated PTCs in diagnosing allograft dysfunction has not been reported. This is most likely explained by the limited number of exfoliated PTCs in urine, in addition to their short life span and weak proliferative ability in cell culture [84]. Cell culture may not be the ideal cell isolation technique in assessing kidney transplant graft function. One should consider the possibilities of cellular de-differentiation that may occur as well as the timeliness of diagnoses, such as graft rejection and other events of graft dysfunction, being delayed by requiring cell culture in the transplant setting [84].

Acknowledging that CD13, sodium–glucose linked transporter-2 and angiotensinogen are expressed as phenotypic markers, our group successfully extracted exfoliated PTCs from urine through specific antibodies and magnetic beads selection methodology [30]. We have employed differential multispectral auto-fluorescence imaging techniques on urinary exfoliated PTCs to explore their potential as a non-invasive early diagnostic tool in CKD [30, 90]. Cell auto-fluorescence originates from native fluorophores (collagen, elastin, tryptophan, reduced levels of nicotinamide adenine dinucleotide and flavins) that play important roles in cell and tissue metabolism [91, 92]. The use of multispectral microscopy can collect native emission data across a broad range of excitation wavelengths. Cell auto-fluorescence features which define each cell’s spectral profile, including parameters from average channel intensity, channel intensity ratio, pixel standard deviations to skewness can be obtained via multispectral microscopy [93]. This may provide a fingerprint that can be used to distinguish the cellular and metabolic characteristics of each cell, from their cell cycle stage, inflammatory state, extent of oxidative stress, and degree of senescence [92, 94–97].

In comparison to CKD, cell exfoliation within the context of kidney transplantation is much less well studied at present. There was a recently published report describing the assessment of urinary exfoliated kidney cells in kidney transplant recipients [98]. In a study involving 12 circulatory death donor kidney transplant recipients, Pizzuti and colleagues aimed to characterize the phenotype and potential applications of urine-derived renal epithelial cells (URECs) that were voided post-transplant [98]. The URECs that are frequently obtained in the early stages following kidney transplantation have been shown to conventionally be derived from proximal tubules [98]. Voided URECs had high proliferating and inflammatory properties, suggesting their potential role in prognosticating post-transplant ischemia–reperfusion and acute kidney injury (AKI) states as well as their immunomodulatory potential [98]. Otherwise, there was also a study by Goerlich and colleagues which aimed to determine whether the quantification of different urinary cells would allow for non-invasive detection of post-transplant graft rejection [31]. The investigators measured urinary cell numbers of CD4 + and CD8 + *T* cells, monocytes/macrophages, urinary exfoliated tubular epithelial cells (TECs), and podocalyxin-positive (PDX +) cells using flow cytometry and correlated their findings to biopsy reports. It was found that combining the amount of urinary *T* cells and TECs, or *T* cells and PDX + cells, demonstrated significant differentiation between patients with graft rejection from those without (both *p* < 0.01, AUC 0.90 and 0.89 respectively) [31]. Therefore, the combination of urinary *T* cells and TECs or urinary *T* cells and PDX + cells may be a useful resource for non-invasive detection of post-transplant graft rejection. This study suggests urinary cell populations analyzed by flow cytometry have the potential to introduce novel non-invasive monitoring methods for kidney transplant recipients.

Recognizing prior encouraging early findings within the CKD context, one would be reasonable to anticipate that exfoliated PTCs could be utilized to detect tubulointerstitial inflammation in transplanted kidneys, with tubulointerstitial inflammation being a histopathological hallmark of acute graft rejection [99]. It is expected that numerous inflammatory cells are exfoliated in urine, given the pathophysiological release of inflammatory triggers during acute graft rejection [100]. While macrophage markers based on the expression of F4/80 and CD11b could be used to detect the level of urinary exfoliated inflammatory cells in this scenario, an optimal method to determine the number and composition of exfoliated PTCs in acute graft rejection and whether they display any associations with histopathological severity (according to the Banff classification) remains to be seen [99, 101, 102]. Similarly, we may hope that urinary exfoliated PTCs can consistently demonstrate potential as a tool to identify events of post-transplant ATN, classically defined by hypoxic injury to the newly transplanted graft tissue and is responsible for up to 90% of acute kidney injury episodes in the first few weeks following kidney transplantation [103]. Over a longer period of post-transplant follow-up, urinary exfoliated PTCs may also be useful in monitoring the progression of epithelial-to-mesenchymal transition toward tubulointerstitial fibrosis, where this can reflect chronic graft rejection and/or the occurrence of post-transplant immunosuppression complications, such as calcineurin toxicity, BK virus nephropathy, and other potential causes of graft dysfunction [104, 105]. Furthermore, there are potential implications of utilizing urinary exfoliated PTCs and other exfoliated kidney cells as a biomarker to monitor resolution of graft rejection and recovery from graft injury, hence avoiding the need for follow-up biopsies to be undertaken in assessing treatment success.

## Opportunities for artificial intelligence-based analysis of urinary exfoliated kidney cells in kidney transplantation

**Fig. 1:**
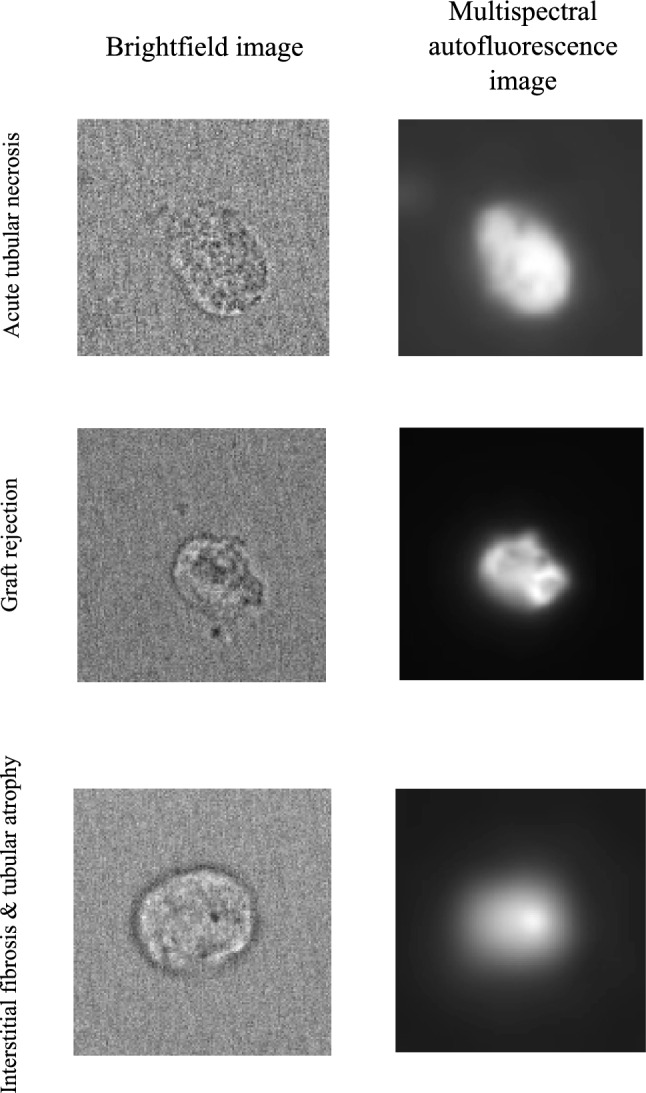
Brightfield images and example multispectral auto-fluorescence images from selected urinary exfoliated PTCs across the ATN, graft rejection and IFTA groups

**Fig. 2:**
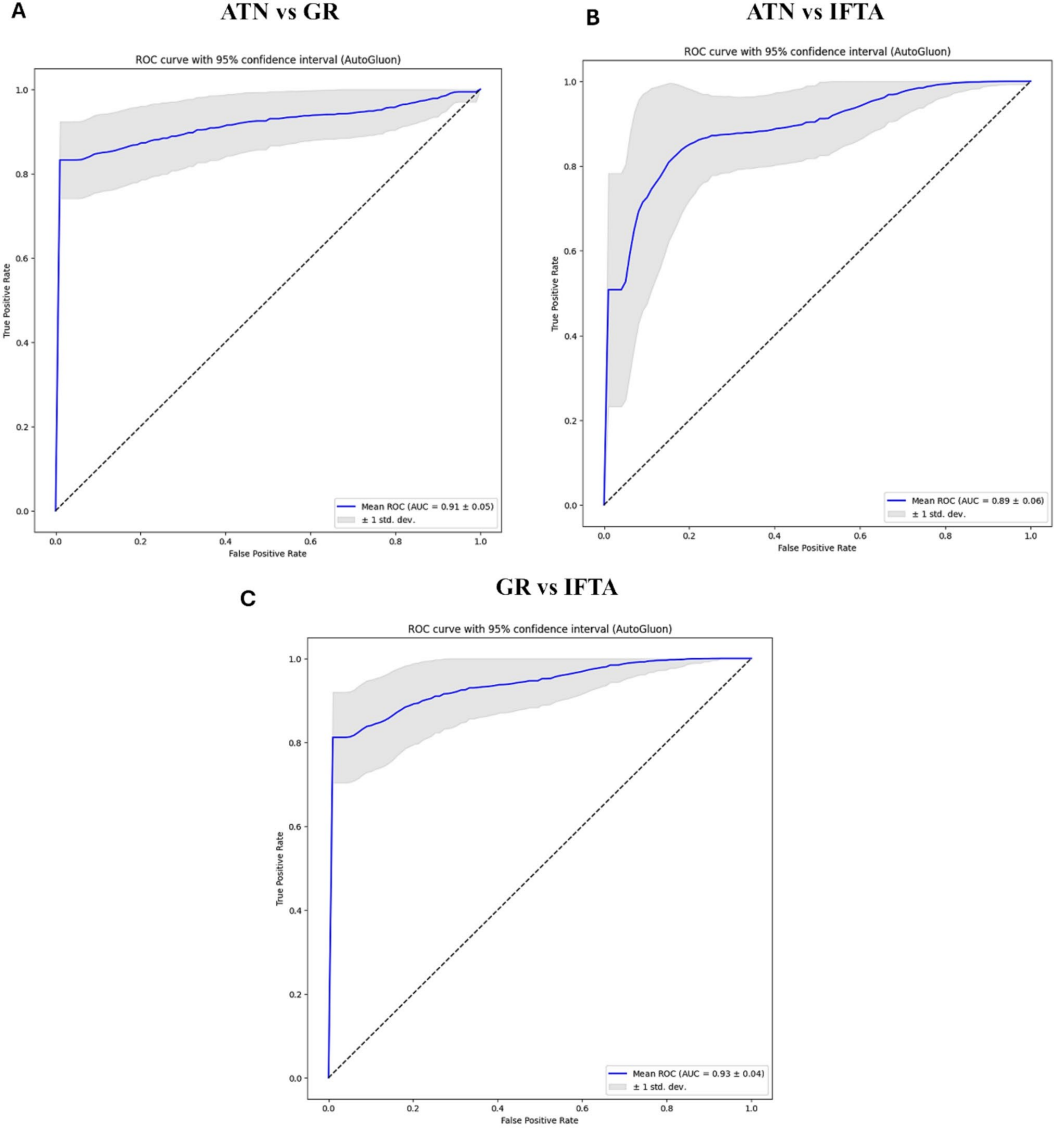
Classification performance between multispectral auto-fluorescence signals of urinary exfoliated PTCs in 57 kidney transplant recipients with post-transplant complications (19 with ATN, 19 with graft rejection, 19 with IFTA). **A** ROC curve with 95% confidence interval for classification between ATN and graft rejection using the AutoGluon framework and the eight top-ranked multispectral auto-fluorescence features. The blue line represents the mean ROC and the gray band shows the variation in performance across resampling runs (AUC 0.91 ± 0.05); **B** ROC curve with 95% confidence interval for classification between ATN and IFTA using the same analysis framework (AUC 0.89 ± 0.06); **C** ROC curve with 95% confidence interval for classification between graft rejection and IFTA using the same analysis framework (AUC 0.93 ± 0.04); *The dashed diagonal line in each panel is the performance of a noninformative classifier (AUC 0.5)*.AUC: Area under the curve; ROC: Receiver operating characteristic; ATN: Acute tubular necrosis; IFTA: Interstitial fibrosis and tubular atrophy

The advent of machine learning and subsequently artificial intelligence (AI) may bring enormous opportunities in kidney transplantation [106]. Other than its original uses aiming to improve radiological and histopathological evaluation of the allograft, machine learning and AI have been increasingly applied for the evaluation of urinary biomarkers to prognosticate graft function and survival, to diagnose events of graft rejection, and to optimize post-transplant immunosuppression [38, 107, 108]. In an era of personalized medicine, AI allows for improved computer-aided diagnostics and quantifiable personalized predictions to achieve more effective patient-orientated care, and machine learning techniques provide increased automation leading to faster evaluation and standardization, generating better analytical performances compared to traditional statistical methods [107, 109, 110]. We have developed a novel diagnostic methodology based on machine learning assessment of urinary exfoliated PTCs multispectral auto-fluorescence signals to accurately discriminate between the causes of various post-transplant complications (Patent ID: AU2024904153) (Fig. 1) [32]. In a pilot study involving 30 patients where we evaluated the ability of our novel methodology, we were able to classify between the post-transplant ATN versus graft rejection, ATN versus interstitial fibrosis and tubular atrophy (IFTA), and graft rejection versus IFTA groups to an excellent degree of discriminative accuracy [32]. We have subsequently extended our analysis to 57 patients total (19 patients in each of the ATN, graft rejection and IFTA comparison groups), where we were able to classify between the post-transplant ATN versus graft rejection, ATN versus IFTA, and graft rejection versus IFTA groups to an excellent degree of discriminative accuracy, with AUC values of 0.91, 0.89 and 0.93 respectively (Fig. 2).

While our early results applying machine learning-based analysis of urinary exfoliated PTCs here showed promise, continued research advancements on integrating machine learning and developing AI platforms for effective assessment of urinary exfoliated PTCs and other kidney cells in the kidney transplantation context are required. One of the challenges to address is whether the application of AI technologies can minimize the number of urinary exfoliated PTCs required in generating useful clinical information for patient management, considering the frequent shortcomings of urinary sources containing enough viable exfoliated PTCs for satisfactory analysis [33].

Going forward from a technical perspective, AI pipelines involving analysis of urinary exfoliated kidney cells would require establishment of a standardized urine collection and processing protocol, reproducible cell enrichment and phenotyping, fixed imaging acquisition settings, transparent feature engineering (intensity statistics, cross-channel ratios and texture measures to capture within-cell heterogeneity) followed by model development with safeguards against overfitting (class imbalance handling, nested cross-validation and leakage controls), and evaluation beyond group-to-group discrimination (calibration and clinically meaningful decision thresholds). Considering the small sample sizes of early studies, external validation and transparent reporting are of utmost importance. Prediction and diagnostic accuracy reports should follow TRIPOD + AI and STARD-AI standards [111, 112]; and clinical trials where there is implementation of AI analysis should follow the CONSORT-AI and SPIRIT-AI standards [113, 114].

## Future directions to improve utilization of urinary exfoliated kidney cells in kidney transplantation

Considering the wide range of potential post-transplant complication’s they could be reflecting on, exfoliated kidney cells appear to be non-specific biomarkers with respect to their pathophysiological formation. For urinary exfoliated kidney cells to take the next step and be translatable as a tool utilized in clinical practice, further investigations are necessary to provide more explicit classification on how exfoliated kidney cells can reliably differentiate between various post-transplant complications. Other than assessing its correlation with kidney histology (which despite being the gold standard is still subjective to interobserver variability), it would also be worthwhile to focus on correlations between the properties of different urinary exfoliated kidney cell types with traditional biomarkers of kidney function (i.e., serum creatinine) as well as the more recently described potential biomarkers of kidney function (i.e., omics biomarkers). Characterizing urinary exfoliated kidney cells by omics technologies will be useful to confirm their specificity compared to kidney histology. Comparing the diagnostic and prognostic accuracy of urinary exfoliated kidney cells to other potential biomarkers may inform us better whether urinary exfoliated kidney cells can be considered the optimal biomarker option within the post-transplant setting.

While acknowledging these physiological and translational constraints on implementing urinary exfoliated kidney cells-based biomarkers in practice, we envision urinary exfoliated kidney cells-based biomarkers primarily as a triage tool to augment current standard-of-care monitoring for suspected transplant graft dysfunction. The short-term goal of utilizing these assays is to focus on improving risk stratification for informing clinical decisions on whether to pursue (or prioritize) transplant biopsy, rather than to outright replace biopsy given the current need for histological phenotyping and the inherent heterogeneity of pathophysiological mechanisms in relation to post-transplant complications. Perhaps urinary exfoliated kidney cells-based biomarkers could also be potentially utilized to predict kidney transplant outcomes prospectively over the medium to longer term. Further studies are required to determine this.

## Conclusion

This article reviewed the evidence and limitations of currently available urinary biomarkers and proposed the increased application of utilizing urinary exfoliated kidney cells including urinary exfoliated PTCs to prognosticate kidney transplant outcomes and to monitor for post-transplant complications. Ultimately, there remains limited evidence on the use of urinary exfoliated kidney cells in the assessment of subjects developing allograft injury post kidney transplantation at present. We hope further advancements, if achieved, could broaden the attraction of employing urinary exfoliated kidney cells for diagnostic and prognostic purposes in kidney transplant management.
